# Author Correction: The role of oxygen-permeable ionomer for polymer electrolyte fuel cells

**DOI:** 10.1038/s41467-023-40630-1

**Published:** 2023-09-01

**Authors:** Ryosuke Jinnouchi, Kenji Kudo, Kensaku Kodama, Naoki Kitano, Takahisa Suzuki, Saori Minami, Kazuma Shinozaki, Naoki Hasegawa, Akihiro Shinohara

**Affiliations:** Toyota Central R&D Labs., Inc, Nagakute, Aichi Japan

**Keywords:** Fuel cells, Fuel cells

Correction to: *Nature Communications* 10.1038/s41467-021-25301-3, published online 16 August 2021

In this article the units on the y-axis of Figures 4a and 6a contained an error. In figure 4a, the y-axis units ‘cm^2^ atm A^−1^’ were incorrectly written as ‘cm^2^ atm mA^−1^’. In figure 6a, the y-axis ‘Specific activity at 0.9 V (mA cm^−2^)’ was incorrectly written as ‘Specific activity at 0.9 V (A cm^−2^)’. The legend for Fig. 4a inadvertently reported the wrong temperature. ‘333 K’ was incorrectly written as ‘353 K’. The original article has been corrected.

The units in the Supplementary Table S2 of this article contained an error in which ‘kcal·mol^−1^·rad^−2^’ was in incorrectly written as ‘kJ·mol−1·rad−2’. The HTML has been updated to include a corrected version of the Supplementary Information.

### Supplementary information

Updated Supplementary Information

