# Supplementary material for: The role of oxygen-permeable ionomer for polymer electrolyte fuel cells
Source: Nat Commun. 2021 Aug 16;12:4956. doi: 10.1038/s41467-021-25301-3 (PMC8368003; doi:10.1038/s41467-021-25301-3)
Supplement: Supplementary file 1 — Supplementary Information [file 41467_2021_25301_MOESM1_ESM.pdf]

Supplementary Information for

The role of oxygen-permeable ionomer for polymer electrolyte fuel cells

Ryosuke Jinnouchi[1], Kenji Kudo[1], Kensaku Kodama[1], Naoki Kitano[1], Takahisa Suzuki[1], Saori Minami[1], Kazuma Shinozaki[1], Naoki Hasegawa[1] and Akihiro Shinohara[1]

[1] Toyota Central R&D Labs., Inc.

Yokomichi 41-1, Nagakute, Aichi 480-1192, Japan

Supplementary Information Section 1 Schematic of HOPI synthesis

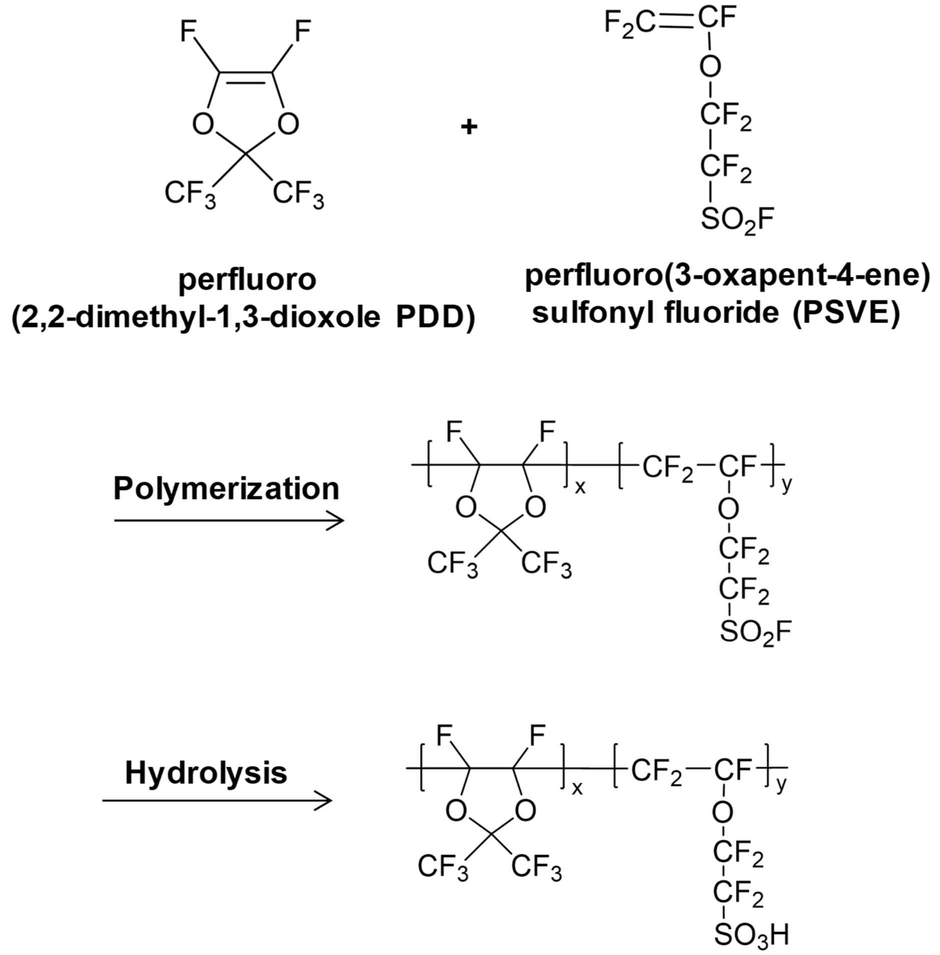

Supplementary Fig. 1: Schematic of HOPI synthesis.

Supplementary Information Section 2 Characterizations of ionomers

The measured EWs of the ionomers were 952 g mol^{-1} (Nafion) and 735 g mol^{-1} (HOPI). For the measurements, the ionomer (10 wt%) was dissolved in a mixture of water and propanol (weight ratio of 3:7). After the solution was homogenized at room temperature, a self-standing ionomer membrane was fabricated by annealing at 413 K for 1 h. The membrane was immersed in a NaCl solution at room temperature overnight. The EW was determined from the pH of the solution after immersion.

The densities were measured as 2.04 g cm^{-3} (Nafion) and 1.93 g cm^{-3} (HOPI) using a gas pycnometer (Ultra Pycnometer 1000, Quantachrome) at room temperature. Before the measurements, samples (approximately 100 mg) were dried in a vacuum chamber at 333 K overnight.

^{19}F-NMR (375MHz) measurement was conducted on samples dissolved in H_2O and 1-propanol (non-deuterated solvents) by using a JEOL JMTC-400/54/SS

spectrometer. The NMR spectra of the Nafion ionomer, HOPI and Aquivion ionomer and their peak assignments are shown in Supplementary Fig. 2**a**. The NMR spectra indicate the presence of both the PFSA and PDD matrices in the HOPI ionomer.

The number-average molecular weight (M_n) of the ionomers was measured by gel permeation chromatography (GPC) (Tosoh HPC-8120GPC) and TSK gel α-M column (Tosoh). As shown in Supplementary Fig. 2**b**, a single peak was observed in the molecular weight distribution curve for each of Nafion and HOPI, and the values of 2.8×10^4 (Nafion) and 3.9×10^4 (HOPI) were obtained. By the GPC result and the NMR spectra, we judge that the HOPI is a co-polymer of the PFSA and PDD. For the measurements, the ionomer (3 wt%) was dissolved in a mixture of water and propanol (weight ratio of 8:2). The solution was homogenized by heating at 513 K for 6 h and was diluted by a dimethyl sulfoxide solution containing 50 mmol L^{-1} LiBr. A molecular weight calibration curve was obtained with pullulan standards (Shodex) in the range of molecular weight of 1420 to 344000 g mol^{-1}.

WAXS patterns shown in Supplementary Fig. 2**c** were recorded on the BL8S3 beamline in Aichi Synchrotron Radiation Center. Ionomer films for the WAXS measurements were prepared from Nafion and HOPI solutions by a solution casting technique. After drying the films at room temperature, they were annealed at 413 K for an hour.

The water uptake λ (the number ratio of water molecules to sulfonate groups) of the ionomers was measured using automated volumetric water adsorption measurement equipment (BELSORP 18, Bell Japan, Inc.) at 298 K. Before the measurements, samples (50 mg) were dried in a vacuum chamber at 353 K for 3 h. The water uptake at the adsorption branch was measured with increasing relative humidity in the sample chamber from 0%RH to 100%RH. At each RH condition, vapour at a given pressure (0.0008 to 0.004 MPa) was dosed to the chamber. The water uptake was determined from the pressure drop caused by the water sorption into the ionomer after confirming that the pressure change in the chamber converged to be less than 1.33 Pa during 600s with each dosing. The pressure after convergence was defined as equilibrium pressure (i.e. RH). The measured water uptake is shown as a function of RH in Supplementary Fig. 3**a**.

The proton conductivity of the ionomers was measured using a chemical impedance meter (Hioki 3532-80) over a frequency range of 1 Hz to 1 MHz at 10–100%RH and room temperature. The results are shown in Supplementary Fig. 3**b**.

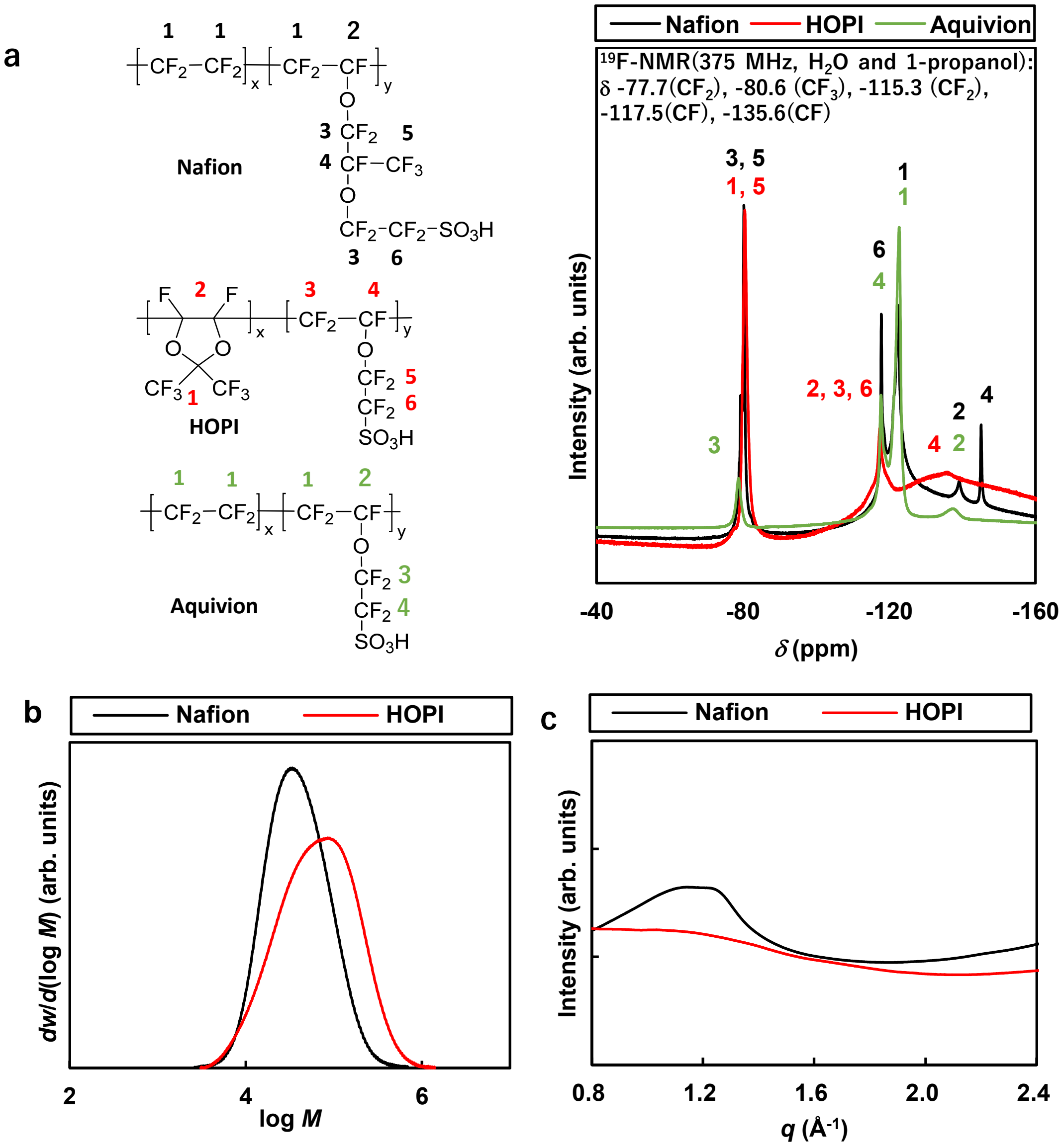

Supplementary Fig. 2: **a**, ^{19}F-NMR spectra of Nafion, HOPI and Aquivion ionomers and peak assignments. **b**, Molecular weight distribution curves obtained by the GPC measurements.**c**, WAXS spectra.

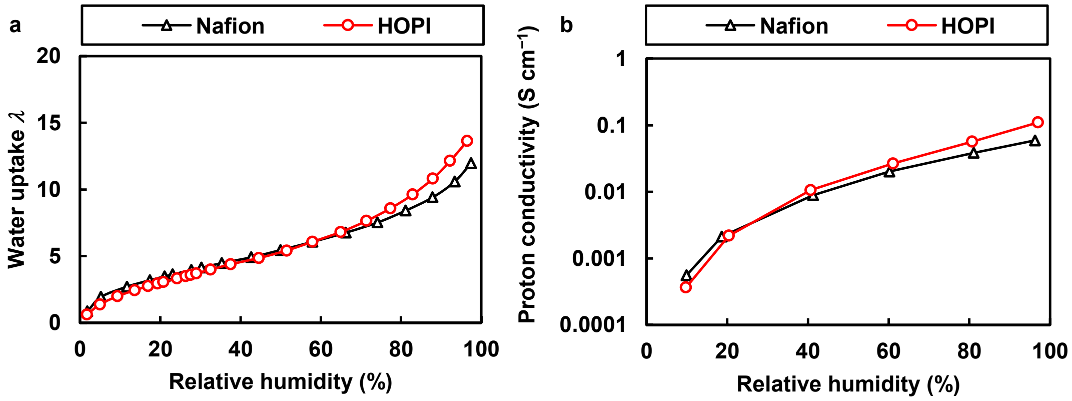

Supplementary Fig. 3: **a**, Water uptake λ and **b**, proton conductivity of the Nafion and HOPI ionomers as a function of RH.

Supplementary Information Section 3 Details of electrochemical characterizations of MEAs

Cell conditioning

Before the measurements, single cells were conditioned by cycling the cell voltage 100 times between OCV and 0.1 V with the sweep rate of 50 mV s^{-1} at 353 K, 0.1 MPa and 90 %RH to assure the stable cell voltage before the measurements (less than 0.3 mV difference between final two cycles). The flow rates of the anode and cathode dry gases were set to 500 and 2000 standard cubic centimetres per minute, respectively, to ensure the reproducibility of the RH and high stoichiometric ratios (35 and 22 at 3.3 A cm^{-2} for the cathode and anode, respectively). To avoid condensations, the gaseous temperature at the inlet of anode and cathode was set to 383 K.

Current–voltage characterizations

The flow rates of the anode and cathode dry gases were equal to those for the cell conditioning. Similar to the conventional protocols in refs. 41, 54 and 55, the current–voltage characteristics of the MEA were measured by varying the cell voltage from 0.3 to 0.9 V at 3 min intervals of 0.1 V at 353 K, 0.1 MPa, and 30, 60, and 90% RH. Current was averaged over the 3 min potential hold. In addition, in order to evaluate the catalytic activity for ORR, current-voltage characteristics at a low current density region were measured by sweeping the voltage at 20 mV s^{-1} after confirming that the potentiodynamic measurement provided the results close to the quasi-steady state results as shown in Supplementary Fig. 4. Pure hydrogen and air were introduced

into the anode and cathode, respectively. The high-frequency resistance was simultaneously measured using an AC impedance meter (FC-100R, Chino Co., Tokyo, Japan) at 10 kHz and used for IR correction. Supplementary Fig. 5 shows Tafel plots of the IR-corrected voltage versus the current per mass of Pt.

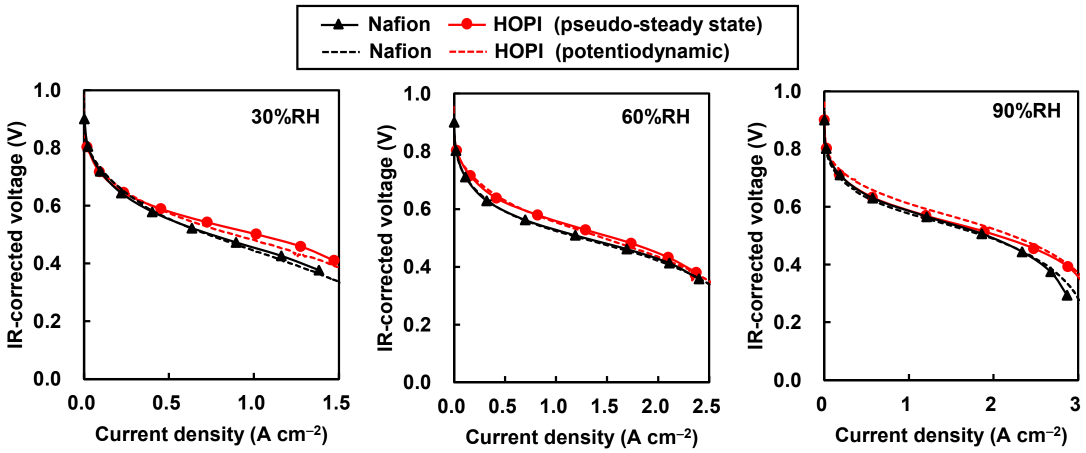

Supplementary Fig. 4: Current-voltage curves obtained by the pseudo-steady state and potentiodynamic measurements with 20 mV s^{-1} sweep rate (cathodic scan). Results for the HOPI and Nafion ionomer are shown in red and black, respectively. The solid lines with circles and triangles show the results by the pseudo-steady state measurements, and the dashed lines show the results by the potentiodynamic measurements.

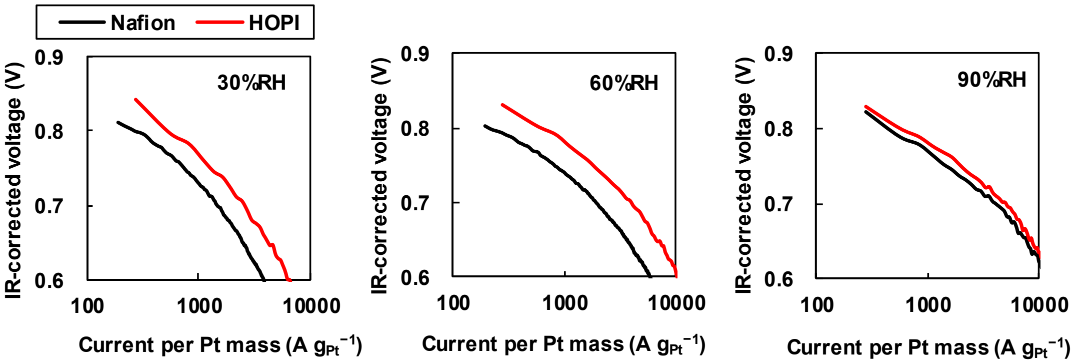

Supplementary Fig. 5: Tafel plots of IR-corrected voltage versus current per Pt mass in MEAs at 353 K and 0.1 MPa (air).

Conditions for measurements of the local oxygen transport resistance

The flow rates of the anode and cathode dry gases were equal to those for the measurements of the current–voltage curves. The absolute total pressure p was controlled to 0.1, 0.13, 0.16, and 0.19 MPa by back-pressure regulators. The H_2 volumetric fraction of the anode dry gas was fixed to 20 % by mixing N_2 gas to suppress the effects of H_2 crossover. The oxygen volumetric fraction in the cathode dry gas was fixed to 1% by mixing N_2 gas to remove the effects of flooding and minimize the effects of ohmic loss. The total flow rates of the anode and cathode dry gases were set to 500 and 2000 standard cubic centimetres per minute, respectively. The partial pressures of the gases at the inlet were calculated from the flow rates of the gases, water vapor pressure, and total pressure. Supplementary Fig. 6 shows the current–voltage curves at 353 K and 30% RH, which were used to calculate the local oxygen transport resistance R_{other} of the Nafion ionomer and HOPI. From the limiting current densities shown in Supplementary Fig. 6**a** and **b**, the total oxygen transport resistance R_{total} was calculated using equation (1). The local oxygen transport resistance R_{other} was determined as the intercept of the plots of R_{total} versus the total pressure p shown in Supplementary Fig. 6**c**.

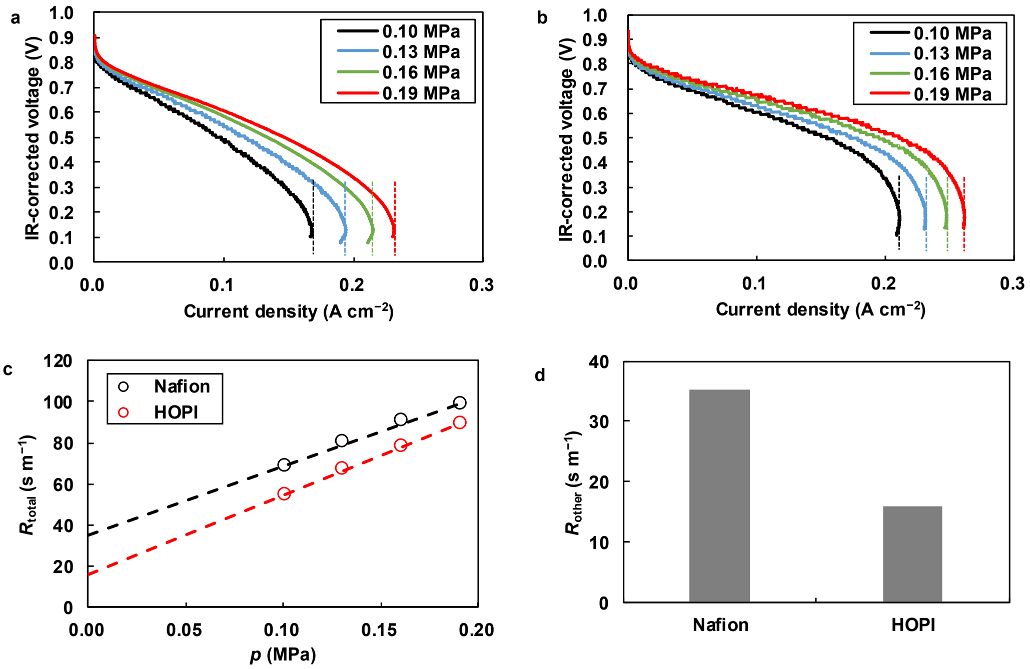

Supplementary Fig. 6: Derivation of local oxygen transport resistance in MEAs. **a** and **b**, Current–voltage curves of the MEAs with Nafion ionomer and HOPI, respectively, at 353 K, 30% RH, and 1% oxygen volumetric fraction. **c**, R_{total} calculated from the limiting current density in **a** and **b** as a function of total pressure p. **d**, R_{other} determined from the intercepts in **c**.

Ohmic resistance of CLs

The ohmic resistance of the CLs was measured by electrochemical impedance spectroscopy to identify the effects of the difference in proton conductivity between the Nafion ionomer and HOPI in the MEAs. The measurements were performed using an electrochemical test system (1280Z, Solartron Analytical) in a frequency range of 1–20,000 Hz with an AC amplitude of 5 mV. The voltage was set to 0.35 V. Pure nitrogen was introduced into the cathode, and the partial pressure of hydrogen was decreased to 0.02 MPa by mixing N_2 to suppress the effects of H_2 crossover. The measured impedance spectra are shown in Supplementary Fig. 7. Following the method developed in ref. 57, the ohmic resistance contribution, $1/3R_{CL}L$, of the CL was determined as the difference in real impedance ReZ between the intercept with the real axis and the inflection point in the spectrum. Here, R_{CL} and L are the ohmic resistance and thickness of the CL, respectively.

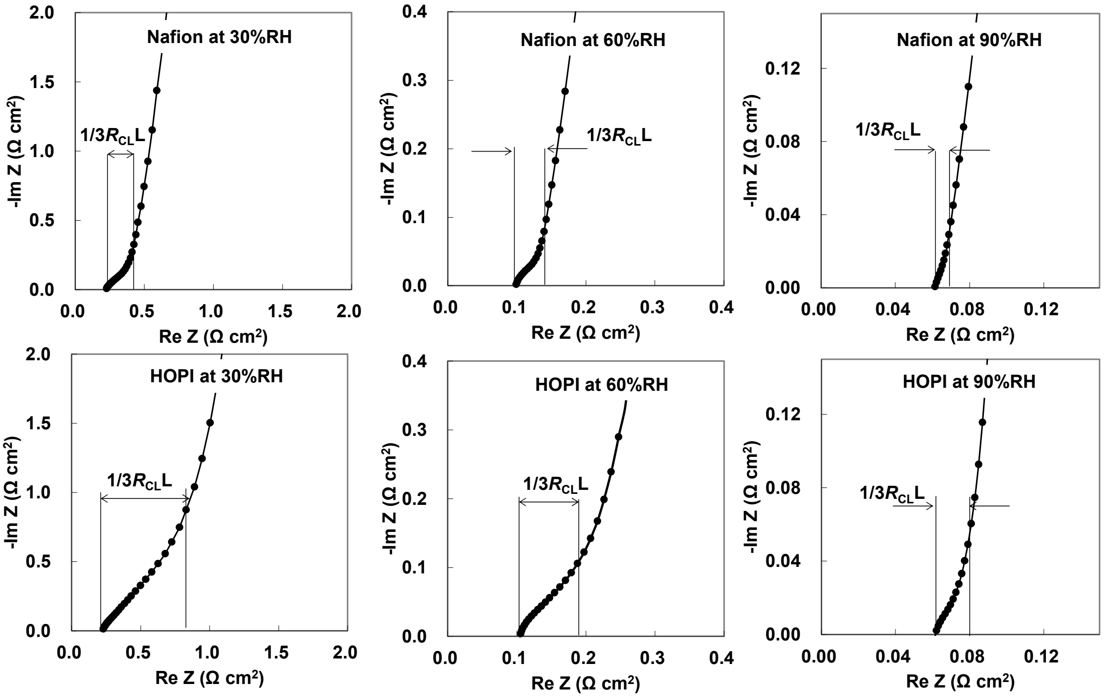

Supplementary Fig. 7: AC impedance spectra and ohmic resistance of CLs ($1/3R_{CL}L$).

Supplementary Information Section 4 Microeletrode techniques: sample preparation and measurements

Ionomer thin films with a thickness of 20–160 nm were prepared on the working Pt disk microelectrodes with diameters of 10 μm by solution casting of 0.3–0.7 wt% Nafion or HOPI solutions diluted by 1-propanol. After drying at room temperature, they were annealed at 403 K for 1 h. The ionomer thickness was measured using a surface profile measurement system (Dektak, Veeco Instruments) under N_2 flow by scratching the film after the electrochemical measurements. The working microelectrode was set in the cell so that the ionomer thin film was in contact with a platinized Pt mesh coated with Nafion. The reference electrode was also comprised of a platinized Pt mesh coated with Nafion and was placed in another compartment supplied with humidified 2% (in dry base) H_2 gas (N_2 balance). Further details of the cell are written in ref. 21. After conditioning the working electrode by cycling the potential between 0.05 and 1.1 V (vs. RHE) 100 times with a sweep rate of 1 V s^{-1} at 90%RH, the limiting current density was measured by sweeping the potential from 0.1 to 1.1 V (vs. RHE) at a sweep rate of 0.1 V s^{-1} and an oxygen concentration of 0.1% (N_2 balance) at 333 K and 30, 60, and 90% RH. The temperature of 333 K was chosen to reduce the effects of contamination. The measured ORR current densities are shown in Supplementary Fig. 8. The limiting current density was determined as the maximum current density in the anodic scan. The inverse of the measured limiting current density is plotted as a function of ionomer thickness in Fig. 4**a**. The interfacial resistance (R_{in}) was determined as the intercept of the linear function fitted to the measured data.

The same cell was used for the measurements for the thick films. An ionomer thick film with a thickness of 100 μm under the humidified condition was prepared on a petri-dish by casting the ionomer solution. The fabricated thick film was cleaned by sequential soaking in 3% H_2O_2 aqueous solution, Millipore ultrapure water, 2 mol L^{-1} HNO_3, and ultrapure water, in that order, at 353 K for 1 h each. The working Pt disk microelectrode with a diameter of 50 μm was set on the thick film. The measurement was carried out after conditioning the working electrode by cycling the potential similarly to the thin film measurement. The transitional ORR limiting current density was measured after the electrode potential was stepped from 1.1 to 0.4 V (vs. RHE) under humidified pure oxygen at 353 K and 30, 60, and 90%RH. Supplementary Fig. 9 shows the transitional limiting current density as a function of $1/\sqrt{t}$. The diffusion coefficients and solubilities of oxygen were determined by fitting the Cottrell-type equation [equation (3)] to the data. The optimized Cottrell-type equation excellently reproduces the measured current density as shown in Supplementary Fig. 9, and therefore, uncertainty caused by

the fitting error is small (less than 10%).

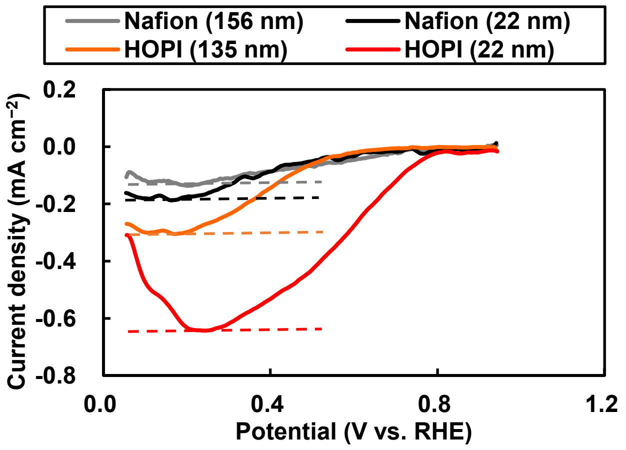

Supplementary Fig. 8: ORR current densities of the ionomer thin films measured at 333 K and 30%RH.

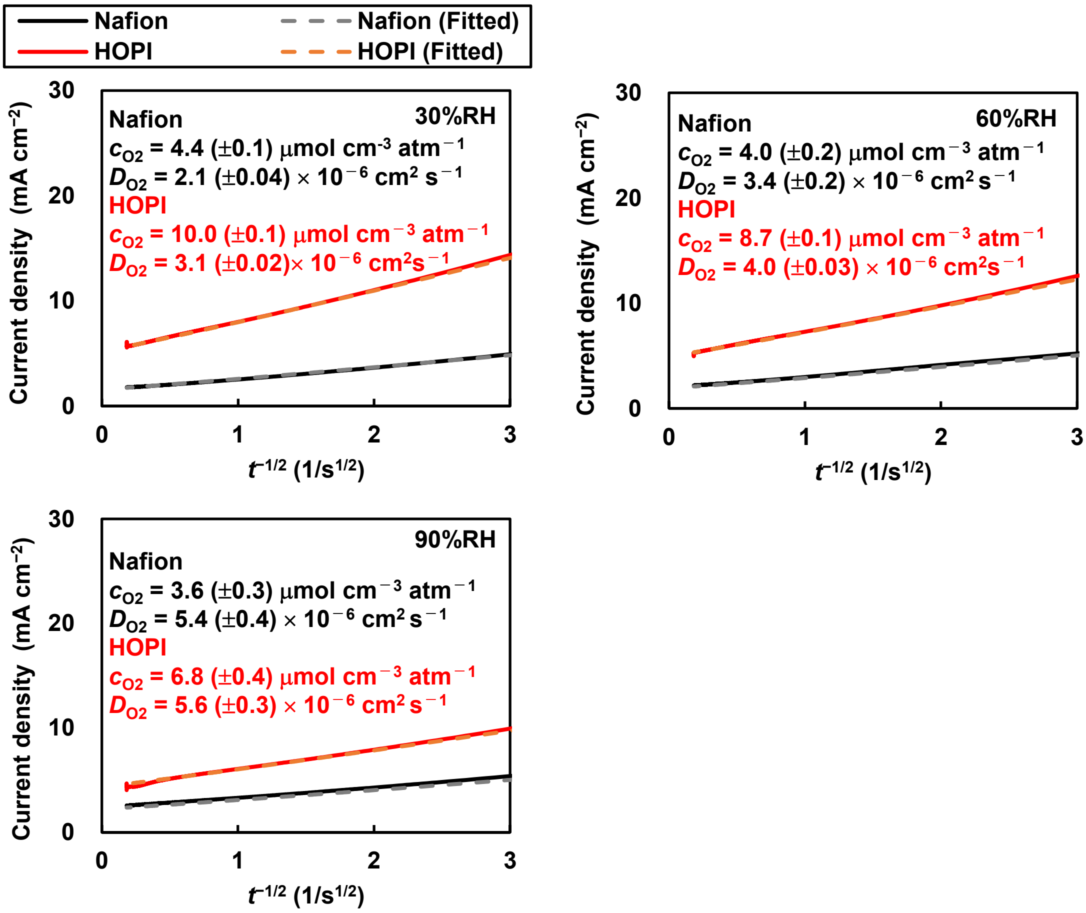

Supplementary Fig. 9: Transitional limiting current density of the ORR measured by the microelectrode technique using ionomer thick films at 353 K and 30, 60, and 90% RH as a function of $1/\sqrt{t}$. The values shown in the parentheses indicate the uncertainty caused by the fitting error, which can be estimated by adopting error propagation inequalities: $\Delta D_{O_2} < \Delta j_L/(\partial j_L/\partial D_{O_2})$ and $\Delta c_{O_2} < \Delta j_L/(\partial j_L/\partial c_{O_2})$. Here, Δj_L indicates the error in the fitted current density.

Supplementary Information Section 5   Models for molecular dynamics simulations

Molecular models of the Nafion ionomer and HOPI are shown in Supplementary Fig. 10. One Nafion ionomer molecule was modelled by connecting six monomers. One HOPI ionomer molecule examined in this study was modelled by connecting seven monomers. The resulting molecular weights and EWs of the Nafion and HOPI molecules are 6903 and 7109, respectively, and 1150 and 1015 g mol^{-1}, respectively. In the simulations, we set the EW of HOPI close to that of Nafion to separately examine the effects of the difference in the backbone and sidechain. The ionomer thin films were modelled by placing two ionomer molecules and water molecules on a Pt(111) surface modelled as a three-layer slab with a two-dimensional periodicity of $8 \times 5\sqrt{3}$ as shown in Supplementary Fig. 11**a**. The number of water molecules was set to give a water content λ of 0, 6, or 11, where λ is defined as the number of water molecules per sulfonate anion. As shown in Supplementary Fig. 3**a**, the water contents of 0, 6, and 11 correspond approximately to 0, 60, and 90% RH, respectively. To avoid molecular diffusions to the infinitely spread vacuum region, a harmonic wall potential was located on top of the high pressure oxygen gas similarly to the previous study. In addition to the ionomer/Pt interfaces, the bulk ionomers were also simulated to compare the theoretical and experimental densities and oxygen diffusion coefficients. The bulk ionomers were modelled as mixtures of ten oxygen molecules, water molecules and ten polymers per unit cell with a three-dimensional periodic boundary condition as shown in Supplementary Fig. 11**b**. For computations of the oxygen solubility, the ionomer thin films isolated in a high pressure pure oxygen gas (20 MPa) was used. As shown in Supplementary Fig. 11**c**, the thin films were modelled by unit cells with a two-dimensional periodicity containing mixtures of four ionomer molecules and water molecules. In the simulations on both bulk and thin film, the number of water molecules was set to give a water content λ of 0, 6, or 11 similarly to the ionomer/Pt interfacial models. Harmonic wall potentials were also located in the thin film models to avoid molecular diffusions to the infinitely spread vacuum region. The parameters of the interatomic potential models for the Nafion ionomer are the same as those in ref. 24. We have optimized the intra-molecular bonding, bending and torsion parameters of the HOPI ionomer to reproduce the structures, energies, and vibrational frequencies of the HOPI monomer obtained by density functional theory calculations. The parameters are listed in Supplementary Tables 1 to 3. Other parameters for the HOPI are equal to those for Nafion.

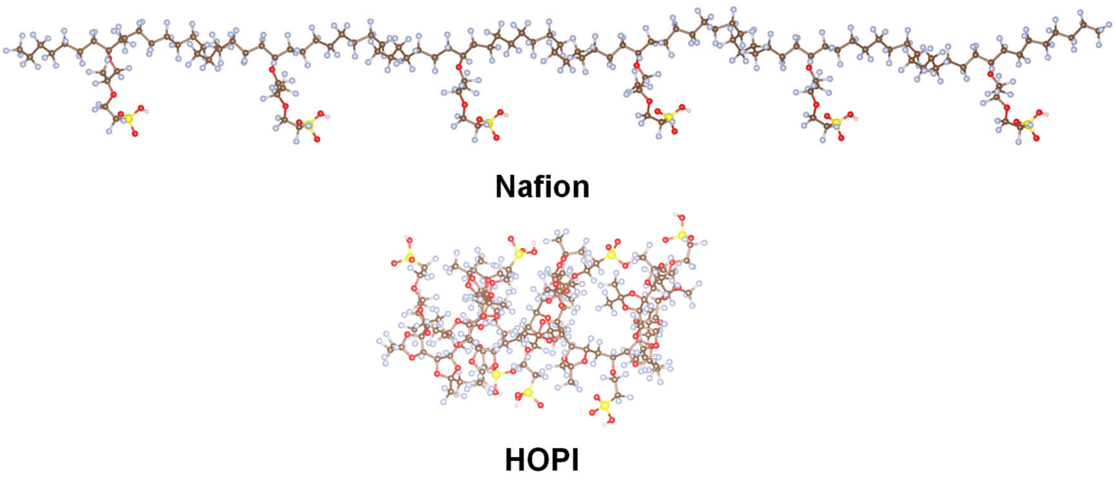

Supplementary Fig. 10: Molecular models of Nafion and HOPI.

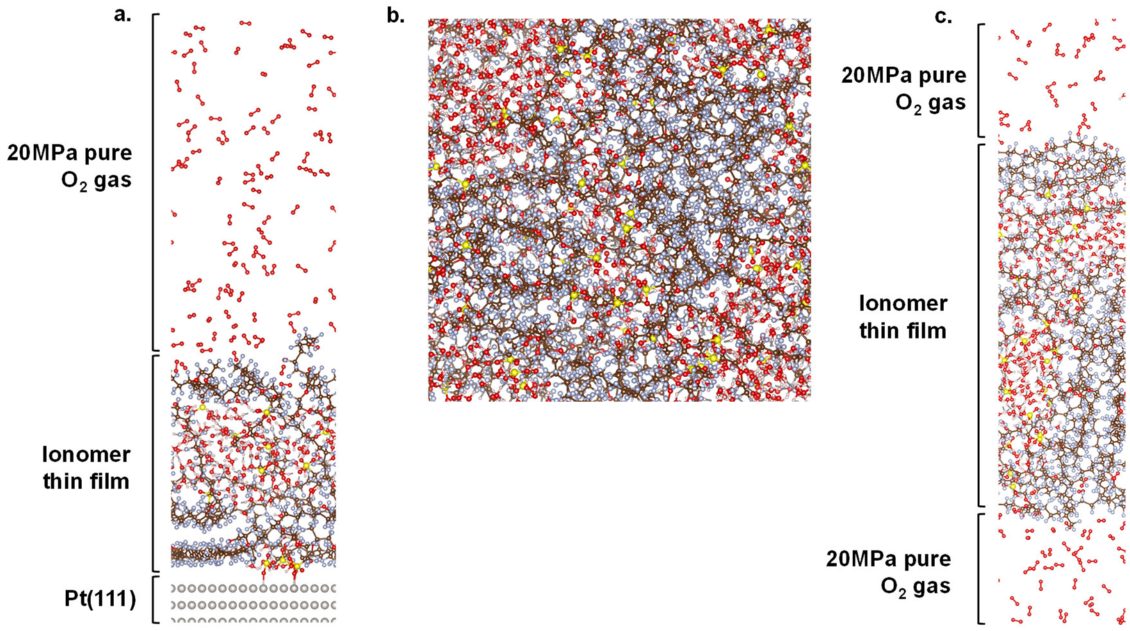

Supplementary Fig. 11: MD simulation models. **a**, Ionomer/Pt interfacial model, **b**, bulk ionomer model, and **c**, ionomer thin film model.

Supplementary Table 1: Intra-molecular bond parameters for the HOPI ionomer. A(B) (B = SO_3^- or SO_3H) shown in the column of “Bond type” indicates an element A in (or linked to) a group B. O_a and O_b indicate oxygen atoms non-bonded and bonded with proton, respectively. The parameters are for two-body bond interactions modelled by $k_{2bond}(r_{ij}-r_{bond}^0)^2+k_{3bond}(r_{ij}-r_{bond}^0)^3+k_{4bond}(r_{ij}-r_{bond}^0)^4$, where r_{ij} denotes the distance between atoms i and j. See further details of the definition in ref. 24.

Bond type	r_{bond}^0 (Å)	k_{2bond} (kJ·mol^{-1}·Å^{-2})	k_{3bond} (kJ·mol^{-1}·Å^{-3})	k_{4bond} (kJ·mol^{-1}·Å^{-4})
C-C	1.500	1249.898	−2499.795	2916.428
C-C(SO_3^-)	1.500	1249.898	−2499.795	2916.428
C-F	1.335	1231.001	−2462.003	2872.337
C(SO_3^-)-F	1.335	1231.001	−2462.003	2872.337
C-O	1.372	1179.260	−2358.520	2751.606
C(SO_3^-)-S(SO_3^-)	1.694	931.742	−1863.484	2174.065
C(SO_3H)-S(SO_3H)	1.946	418.500	−837.000	976.500
H(SO_3H)- O_b(SO_3H)	0.960	2141.117	−4282.233	4995.939
S(SO_3^-)-O(SO_3^-)	1.474	2303.706	−4607.413	5375.315
O_a(SO_3H)-S(SO_3H)	1.464	1663.203	−3326.405	3880.806
O_b(SO_3H)-S(SO_3H)	1.464	1663.203	−3326.405	3880.806

Supplementary Table 2: Intra-molecular angular parameters for the HOPI ionomer. The parameters are for three-body angular interactions modelled by $k_{2\text{bend}}(\theta_{ijk}-\theta_{\text{bend}}^0)^2+k_{3\text{bend}}(\theta_{ijk}-\theta_{\text{bend}}^0)^3+k_{4\text{bend}}(\theta_{ijk}-\theta_{\text{bend}}^0)^4$, where the angle θ_{ijk} denotes the angle $\angle ijk$ of atoms i, j and k. See further details of the definition in ref. 24.

Bend type	θ_{bend}^0 (°)	$k_{2\text{bend}}$ (kcal·mol^{-1}·rad^{-2})	$k_{3\text{bend}}$ (kcal·mol^{-1}·rad^{-3})	$k_{4\text{bend}}$ (kcal·mol^{-1}·rad^{-4})
C-C-C	103.340	21.312	−2.742	−4.416
C-C-F	105.872	71.258	−11.010	−14.904
C(SO_3^-)-C-F	105.872	71.258	−11.010	−14.904
C-C(SO_3^-)-F	105.872	71.258	−11.010	−14.904
C-C-O	118.698	42.113	−12.688	−9.500
C(SO_3^-)-C-O	118.698	42.113	−12.688	−9.500
C-C-S(SO_3H)	103.241	59.477	−7.592	−12.319
C-O-C	110.547	73.191	−14.963	−15.647
C-S(SO_3H)-O_a(SO_3H)	115.101	94.299	−24.207	−20.721
C-S(SO_3H)-O_b(SO_3H)	115.101	94.299	−24.207	−20.721
F-C-F	108.359	127.631	−23.059	−26.986
F-C(SO_3^-)-F	108.359	127.631	−23.059	−26.986
F-C-O	109.875	92.495	−18.227	−19.704
F-C-S(SO_3H)	96.625	10.000	−0.628	−2.038
H(SO_3H)-O_b(SO_3H)-S(SO_3H)	96.975	128.881	−8.529	−26.275
O_a(SO_3H)-S(SO_3H)-O_a(SO_3H)	124.595	31.933	−12.226	−7.593
O_a(SO_3H)-S(SO_3H)-O_b(SO_3H)	124.595	31.933	−12.226	−7.593
C-C(SO_3^-)-S(SO_3^-)	122.960	10.000	−3.590	−2.340
C(SO_3^-)-S(SO_3^-)-O(SO_3^-)	112.527	53.287	−12.078	−11.520
F(SO_3^-)-C(SO_3^-)-S(SO_3^-)	104.367	62.976	−8.757	−13.095
O(SO_3^-)-S(SO_3^-)-O(SO_3^-)	127.152	122.123	−51.580	−29.830
O-C-O	115.817	31.956	−8.478	−7.056

Supplementary Table 3: Intra-molecular torsion parameters for the HOPI ionomer. The parameters are for four-body torsion interactions modelled by $k_{1torsion}(1-\cos\phi_{ijkl})+k_{2torsion}(1-\cos2\phi_{ijkl})+k_{3torsion}(1-\cos3\phi_{ijkl})$, where the angle ϕ_{ijkl} denotes the torsion angle composed of atoms *i*, *j*, *k* and *l*. See further details of the definition in ref. 24.

Torsion type	$k_{1torsion}$ (kJ·mol^{-1})	$k_{2torsion}$ (kJ·mol^{-1})	$k_{3torsion}$ (kJ·mol^{-1})
C-C-C-C	0.000	0.000	−6.691
C-C-C-F	0.000	0.000	0.985
C-C-C(SO_3^-)-F	0.000	0.000	0.985
C(SO_3^-)-C-C-F	0.000	0.000	0.985
F-C-C-F	0.000	0.000	−6.762
F-C-C(SO_3^-)-F	0.000	0.000	−6.762
F-C-S(SO_3H)-O_a(SO_3H)	0.000	0.000	−3.746
F-C-S(SO_3H)-O_b(SO_3H)	0.000	0.000	−3.746
C-C-S(SO_3H)-O_a(SO_3H)	0.000	0.000	−3.746
C-C-S(SO_3H)-O_b(SO_3H)	0.000	0.000	−3.746
F-C-C-S(SO_3H)	0.000	0.000	−23.286
F-C-C-O	0.000	0.000	−9.153
F-C(SO_3^-)-C-O	0.000	0.000	−9.153
F-C-O-C	0.000	0.000	−5.179
C-C-C-O	0.000	0.000	12.491
O-C-C-O	0.000	0.000	−69.921
C-C-O-C	0.000	0.000	8.328
C(SO_3^-)-C-O-C	0.000	0.000	8.328
O-C-C(SO_3^-)-S(SO_3^-)	0.000	0.000	−0.054
O-C-C-S(SO_3H)	0.000	0.000	−33.990
C-C(SO_3^-)-S(SO_3^-)-O(SO_3^-)	0.000	0.000	18.841
F-C-C(SO_3^-)-S(SO_3^-)	0.000	0.000	−6.078
F-C(SO_3^-)-S(SO_3^-)-O(SO_3^-)	0.000	0.000	11.097
C-O-C-O	0.000	0.000	3.466
C-S(SO_3H)-O_b(SO_3H)-H(SO_3H)	0.000	0.000	−4.008
H(SO_3H)-O_b(SO_3H)-S(SO_3H)-O_a(SO_3H)	0.000	0.000	11.587
