# Supplementary material for: The role of oxygen-permeable ionomer for polymer electrolyte fuel cells
Source: Nat Commun. 2021 Aug 16;12:4956. doi: 10.1038/s41467-021-25301-3 (PMC8368003; doi:10.1038/s41467-021-25301-3)
Supplement: Supplementary file 3 — Reporting Summary [file 41467_2021_25301_MOESM3_ESM.pdf]

nature portfolio

Corresponding author(s): Ryosuke Jinnouchi

Last updated by author(s): Jul 1, 2021

Reporting Summary

Nature Portfolio wishes to improve the reproducibility of the work that we publish. This form provides structure for consistency and transparency in reporting. For further information on Nature Portfolio policies, see our Editorial Policies and the Editorial Policy Checklist.

Statistics

For all statistical analyses, confirm that the following items are present in the figure legend, table legend, main text, or Methods section.

n/a	Confirmed	
☐	☒	The exact sample size (*n*) for each experimental group/condition, given as a discrete number and unit of measurement
☐	☒	A statement on whether measurements were taken from distinct samples or whether the same sample was measured repeatedly
☒	☐	The statistical test(s) used AND whether they are one- or two-sided<br>*Only common tests should be described solely by name; describe more complex techniques in the Methods section.*
☒	☐	A description of all covariates tested
☐	☒	A description of any assumptions or corrections, such as tests of normality and adjustment for multiple comparisons
☐	☒	A full description of the statistical parameters including central tendency (e.g. means) or other basic estimates (e.g. regression coefficient) AND variation (e.g. standard deviation) or associated estimates of uncertainty (e.g. confidence intervals)
☒	☐	For null hypothesis testing, the test statistic (e.g. *F*, *t*, *r*) with confidence intervals, effect sizes, degrees of freedom and *P* value noted<br>*Give P values as exact values whenever suitable.*
☒	☐	For Bayesian analysis, information on the choice of priors and Markov chain Monte Carlo settings
☒	☐	For hierarchical and complex designs, identification of the appropriate level for tests and full reporting of outcomes
☒	☐	Estimates of effect sizes (e.g. Cohen's *d*, Pearson's *r*), indicating how they were calculated

Our web collection on statistics for biologists contains articles on many of the points above.

Software and code

Policy information about availability of computer code

Data collection	The molecular dynamics simulations were carried out by using a home-made Fortran program developed internally in Toyota Central R&D Labs., Inc (TCRDL). The program is not commercially available and distributable because of a policy in TCRDL.
Data analysis	Data analysis was done by the standard Microsoft Excel software, which is commercially available widely.

For manuscripts utilizing custom algorithms or software that are central to the research but not yet described in published literature, software must be made available to editors and reviewers. We strongly encourage code deposition in a community repository (e.g. GitHub). See the Nature Portfolio guidelines for submitting code & software for further information.

Data

Policy information about availability of data

All manuscripts must include a data availability statement. This statement should provide the following information, where applicable:

- Accession codes, unique identifiers, or web links for publicly available datasets
- A description of any restrictions on data availability
- For clinical datasets or third party data, please ensure that the statement adheres to our policy

All data are available in the main text and Supplementary Information. Additional datasets related to this study are available from the corresponding author upon reasonable request. Source data are provided with this paper.

Field-specific reporting

Please select the one below that is the best fit for your research. If you are not sure, read the appropriate sections before making your selection.

☐ Life sciences ☐ Behavioural & social sciences ☒ Ecological, evolutionary & environmental sciences

For a reference copy of the document with all sections, see nature.com/documents/nr-reporting-summary-flat.pdf

Ecological, evolutionary & environmental sciences study design

All studies must disclose on these points even when the disclosure is negative.

Study description	Analyses combining the electrochemical measurements of single cells, microelectrodes, and single-crystal surfaces with MD simulations elucidated the role of the highly oxygen permeable ionomer in polymer electrolyte fuel cells.
Research sample	Membrane electrode assemblies (MEAs), microelectrodes, and single crystal electrodes with conventional Nafion ionomer and our newly synthesized highly oxygen permeable ionomer. Samples were selected to clarify two roles of our new ionomer by analyses combining single cell, microelectrodes and single crystal electrodes.
Sampling strategy	In measurements on single cell, microelectrodes covered by ionomer thick films and single crystal electrodes, two samples were measured to check the reproducibility. For microelectrodes covered by ionomer thin film, 5 samples were measured changing the ionomer thickness. In molecular dynamics simulations, 2 to 3 simulations were carried out, and obtained properties were averaged.
Data collection	Current-voltage curves of MEAs, microelectrodes, and single crystal electrodes were taken by charge/discharge unit and/or potentiostat by Naoki Kitano, Saori Minami, and Kensaku Kodama. Molecular dynamics simulations were carried out on our internal PC-clusters. Simulations were carried out by Ryosuke Jinnouchi.
Timing and spatial scale	The measurements of the MEAs used in the final manuscript were started from January in 2021 and stopped in May, 2021. The measurements on microelectrodes and single crystal surfaces were carried out from April, 2017 to December, 2017. Molecular dynamics simulations on the Nafion ionomer were carried out from April, 2016 to May, 2021. Simulations on the new ionomer were carried out from 2017, April to May, 2021. All data were taken in Toyota Central R&D Labs., Inc.
Data exclusions	All data were used in the figures presented in our manuscript.
Reproducibility	In measurements on single cell, microelectrodes covered by ionomer thick films and single crystal electrodes, two samples were measured to check the reproducibility. For microelectrodes covered by ionomer thin film, 5 samples were measured changing the ionomer thickness. In molecular dynamics simulations, 2 to 3 simulations were carried out, and obtained properties were averaged.
Randomization	All samples (MEAs and microelectrodes) were prepared to realize high-performance and/or to accurately measure the targeted properties. Therefore, randomization is not taken into account. In the molecular dynamics simulations, initial locations of polymer and molecules were randomly prepared. Initial velocities were randomly set to follow the Boltzmann distribution. Two to three structures were prepared to compute mean physical properties.
Blinding	Blinding was not possible for our study. An experimentalist or theorist who prepared for samples needed to measure the sample by himself/herself because of the limited number of employees in our company.

Did the study involve field work? ☐ Yes ☒ No

Reporting for specific materials, systems and methods

We require information from authors about some types of materials, experimental systems and methods used in many studies. Here, indicate whether each material, system or method listed is relevant to your study. If you are not sure if a list item applies to your research, read the appropriate section before selecting a response.

Materials & experimental systems

n/a	Involved in the study	
☒	☐	Antibodies
☒	☐	Eukaryotic cell lines
☒	☐	Palaeontology and archaeology
☒	☐	Animals and other organisms
☒	☐	Human research participants
☒	☐	Clinical data
☒	☐	Dual use research of concern

Methods

n/a	Involved in the study	
☒	☐	ChIP-seq
☒	☐	Flow cytometry
☒	☐	MRI-based neuroimaging
